# Combination of NKT14m and Low Dose IL-12 Promotes Invariant Natural Killer T Cell IFN-γ Production and Tumor Control

**DOI:** 10.3390/ijms21145085

**Published:** 2020-07-18

**Authors:** Peng Guan, Robert Schaub, Kim E. Nichols, Rupali Das

**Affiliations:** 1 Division of Oncology, Children’s Hospital of Philadelphia, Philadelphia, PA 19104, USA; guanp@email.chop.edu; 2 RGS Consulting, 118 Jeremy Hill Road Pelham, Pelham, NH 03076, USA; rgschaub@aol.com; 3 Department of Oncology, St. Jude Children’s Research Hospital, Memphis, TN 38105, USA; kim.nichols@stjude.org; 4 Department of Physiology, Michigan State University, East Lansing, MI 48824, USA

**Keywords:** invariant natural killer T cells, monoclonal antibodies, NKT14m, IL-12, cancer immunotherapy

## Abstract

Invariant natural killer T (iNKT) cells are innate-like T lymphocytes characterized by the expression of an invariant T cell receptor (iTCR) that recognizes glycolipid antigens presented by the MHC I-like CD1d molecule. Following antigenic stimulation, iNKT cells rapidly produce large amounts of cytokines that can trans-activate dendritic cells (DC) and promote the anti-tumor functions of cytotoxic lymphocytes, such as natural killer (NK) and CD8 T cells. Additionally, iNKT cells can mediate robust and direct cytotoxicity against CD1d^+^ tumor targets. However, many tumors down-regulate CD1d and evade iNKT cell attack. To circumvent this critical barrier to iNKT cell anti-tumor activity, a novel monoclonal antibody (mAb), NKT14 has been recently developed. This agonistic antibody binds directly and specifically to the iTCR of murine iNKT cells. In the current study, we demonstrate that NKT14m mediates robust activation, cytokine production and degranulation of murine iNKT cells, in vitro. Consistently, NKT14m also promoted iNKT cell activation and immunomodulatory functions, in vivo. Finally, administration of NKT14m with low dose interleukin (IL)-12 further augmented iNKT cell IFN-γ production in vivo, and this combination conferred superior suppression of tumor cell growth compared to NKT14m or IL-12 alone. Together, these data demonstrate that a combination treatment consisting of low dose IL-12 and iTCR-specific mAb may be an attractive alternative to activate iNKT cell anti-tumor functions.

## 1. Introduction

Invariant natural killer T (iNKT) cells comprise a unique lineage of innate-type lymphocytes that express natural killer (NK) cell-specific markers as well as a T cell receptor (TCR) [1]. However, unlike conventional CD4^+^ and CD8^+^ T cells that collectively express a wide range of TCRs, each with its own specificity, the majority of human and murine iNKTs express a restricted or “invariant” TCR repertoire [2]. In mice, the restricted TCR α-chain, Vα14-Jα18, often pairs with Vβ8.2, Vβ7 or Vβ2, whereas in humans, Vα24-Jα18 combines with Vβ11 [1,3]. Furthermore, unlike conventional T cells, which recognize peptide antigens presented in complexes with MHC class I or II, iNKT cells recognize glycolipid antigens such as α-galactosylceramide (αGC) [4], presented by the MHC class I-like molecule CD1d. Following invariant TCR (iTCR) engagement, iNKT cells rapidly secrete Th1 and Th2 cytokines [5] and promote the functions of several immune cells including natural killer (NK), T, B and dendritic cells. As such, iNKT cells bridge the innate and adaptive immune responses and regulate host immunity in infection, allergy, autoimmunity and cancer [6].

Many studies implicate iNKT cells as important mediators of anti-tumor immune surveillance. This has been examined most extensively in mice, where iNKT cell deficiency increases susceptibility to spontaneous [7,8], carcinogen-induced [9,10] or adoptively transferred tumors [11], and in some of these models, reconstitution of the iNKT cell compartment prevents or slows tumor formation [9]. Additionally, iNKT cells protect against B cell lymphomas in mice [11] and promote T-cell killing of EBV-positive human tumor cells in a humanized mouse model of EBV infection [12]. In cancer patients, iNKT cell numbers are not only reduced but also impaired in their functions [13]. Conversely, in patients with neuroblastoma [14] or colon cancer [15], the number of tumor-infiltrating iNKT cells often directly correlates with improved survival.

Invariant NKT cells mediate their anti-tumor function in large part via the activation of other cytolytic effectors such as CD8^+^T and NK cells [10,16,17]. Indeed, αGC or cytokine-stimulated iNKT cells robustly produce IFN-γ and up-regulate the expression of CD40 ligand. As a result, they promote dendritic cell (DC) activation and enhance DC-mediated priming of tumor-specific CD4^+^ and CD8^+^T cell responses [18]. Invariant NKT cell-DC interactions also stimulate DC production of interleukin (IL)-12, which serves to further augment NK and CD8^+^T cell lysis of tumors [16]. Mature iNKT cells basally express cytolytic proteins such as perforin and granzyme B [19,20,21] and can be induced to up-regulate death-promoting molecules such as Fas ligand and TRAIL [21,22,23]. Accordingly, they can also mediate direct cytotoxicity against numerous CD1d^+^ tumors in vitro and in vivo [24,25,26,27]. However, many tumors evade or dampen iNKT cell anti-tumor response by downregulating CD1d or by actively suppressing iNKT cell functions.

A clinically viable approach to harness and augment iNKT cell anti-tumor function against various cancers is the development of agonistic monoclonal antibodies. To that end, two novel monoclonal antibodies, NKTT320 [28] and NKT14m [29,30], specific for the iTCR expressed on human and murine iNKT cells, respectively, have been recently described. In the current study, we further characterized the therapeutic potential of NKT14m in vitro and in vivo. Here, we demonstrate that NKT14m not only activates and promotes murine iNKT cell cytokine production, but it also induces robust degranulation in vitro. Additionally, we examined the immuno-stimulatory and anti-tumor effects of NKT14m in vivo. Injection of C57BL/6 (B6) mice with a single dose of NKT14m (15–150 µg) induced robust murine iNKT cell activation, IFN-γ production, and transactivation of other immune cells. As IL-12 potentiates iNKT cell activation and cytolytic activity, we next examined the effect of IL-12 on NKT14m-mediated iNKT cell function. Administration of NKT14m with low dose IL-12 significantly augmented NKT14m-induced cytokine production. Accordingly, treatment of EL4 T-lymphoma-bearing mice with a combination of NKT14m and IL-12 led to significant reduction in tumor growth in vivo. Taken together with prior studies [29,30], these data support the further development of iNKT cell-activating antibodies as a potential alternative for cancer immunotherapy.

## 2. Materials and Methods

### 2.1. Mice

C57BL/6 (B6) mice were purchased from Jackson Laboratories (Bar Harbor, ME, USA) and housed at the Children’s Hospital of Philadelphia (CHOP; Philadelphia, PA, USA) under specific pathogen-free conditions. The Institutional Animal Care and Use Committee at CHOP approved all experimental procedures.

### 2.2. Reagents

Alpha-galactosylceramide (αGC) analogue, PBS44, was a kind gift from Dr. Paul B. Savage (Brigham Young University; Provo, UT, USA). LIVE/DEAD Fixable Aqua Dead Cell dye and recombinant mouse IL-12 and EL4 T lymphoma cells were purchased from Invitrogen (Carlsbad, CA, USA), Miltenyi (Auburn, CA, USA) and American Type Culture Collection (Manassas, VA, USA), respectively. EL4 cells were cultured in RPMI 1640 medium supplemented with 10% fetal calf serum, 2 mM L-glutamine, 100 U/mL penicillin and 100 U/mL streptomycin. NKT14m and isotype antibody were provided by NKT Therapeutics. NKT14m is a mouse IgG2a with point mutations to the Fc portion (L235E, E318A, K320A and K322A) to greatly reduce antibody dependent cellular cytotoxicity and complement-dependent cytotoxicity function [29].

### 2.3. Antibodies and Flow Cytometry

The antibodies used for immunofluorescence staining (TCRβ, CD4, CD8, NK1.1 CD11c, CD25, CD69, IL-4, IFN-γ, B220, CD107a, CD86 and fluorochrome attached isotype matched IgG1 and IgG2b) were purchased from BD PharMingen (San Jose, CA, USA). The catalog number and clone for each of the antibodies used is included in Table S1 (Supplementary Materials). Fluorochrome conjugated CD1d-tetramer (CD1d-Tet) loaded with glycolipid antigen (PBS57) or unloaded controls were provided by the NIH Tetramer Core Facility (Emory University, Atlanta, GA, USA). For staining cell surface molecules, 1–5 × 10^6^ cells were resuspended in 50 µL of FACS buffer (PBS with 1% fetal calf serum) with pre-titrated optimal concentrations of fluorochrome-conjugated monoclonal antibodies specific for cell surface proteins (30 min, 4 °C, in the dark). Subsequently, cells were washed twice with ice-cold FACS buffer. For intracellular staining, cells were resuspended in 200 µL of Cytofix/Cytoperm™ solution (BD Biosciences) for 20 min at 4 °C. Cells were washed twice in 1x Perm/Wash™ solution (BD Biosciences, 1 mL/wash) and then incubated with pre-determined optimal concentrations of fluorochrome-conjugated anti-cytokine antibodies (prepared in 1x Perm/Wash™ solution) at 4 °C for 1 h in the dark. Cells were then washed twice and resuspended in 200 µL of FACS buffer for flow cytometric analysis. Data were collected on a BD LSRII flow cytometer (BD Biosciences) and analyzed using FlowJo software (FlowJo LLC; Ashland, OR, USA).

### 2.4. Isolation of Purified Populations of iNKT Cell

Hepatic mononuclear cells were isolated from the liver using density centrifugation with Percoll, (GE, Piscataway, NJ, USA) stained with NK1.1 and TCRβ antibodies and then sorted for NK1.1^+^TCRβ^+^ cells using BD FACS Aria (BD Biosciences) [27]. Cells obtained were routinely >97% NK1.1+ and TCRβ+ and were largely iNKT cells (>92%), when stained using PBS57-loaded CD1d tetramers.

### 2.5. In Vitro iNKT Cell Activation

Purified liver iNKT cells from B6 mice were cultured on plate-bound NKT14 m (1–20 µg/mL) or isotype control antibody (20 µg/mL) in a total volume of 200 µL for 24 h. Fluorochrome-conjugated CD107a antibody was added directly to the wells. At the end of incubation, cells were harvested and analyzed for cell viability, activation markers (CD25 and CD69) and degranulation (CD107a) using flow cytometry. Culture supernatants were collected and analyzed for cytokines using enzyme-linked immunosorbent assays (ELISA; BD OptEIA™).

### 2.6. In Vivo iNKT Cell Activation

For in vivo iNKT cell activation, mice were injected intraperitoneally (i.p.) with 4 μg of PBS44 or intravenously (i.v.) with NKT14m (15–150 µg) or isotype control antibody (150 µg) in 300 µL of sterile PBS. In some experiments, mice were also injected i.p. with 100 ng of IL-12 alone or in combination with NKT14m or isotype control antibody (i.v., 30 min after IL-12 injection). After 6 h, the serum was collected and analyzed for levels of cytokines using enzyme-linked immunosorbent assays (ELISA; BD OptEIA™). Splenocytes and liver lymphocytes were isolated and stained for expression of cell surface markers or intracellular production of cytokines [27].

### 2.7. In Vivo Tumor Model

B6 mice were engrafted subcutaneously (s.c.) with 3 × 10^5^ EL4 cells in the right flank. Seventy-two hours later, mice were injected i.v. with a single dose of NKT14m with or without IL-12 (100 ng; i.p. or intra-tumor). Tumor growth was measured using a digital caliper every 2–3 days and tumor volume calculated using the formula length × (width)^2^/2.

### 2.8. Statistics

Statistical analyses were performed using GraphPad PRISM software (San Diego, CA, USA). A *p*-value less than or equal to 0.05 was deemed to be significant.

## 3. Results

### 3.1. NKT14m Induces Murine iNKT Cell Activation, Degranulation and Cytokine Production In Vitro

**Figure 1:**
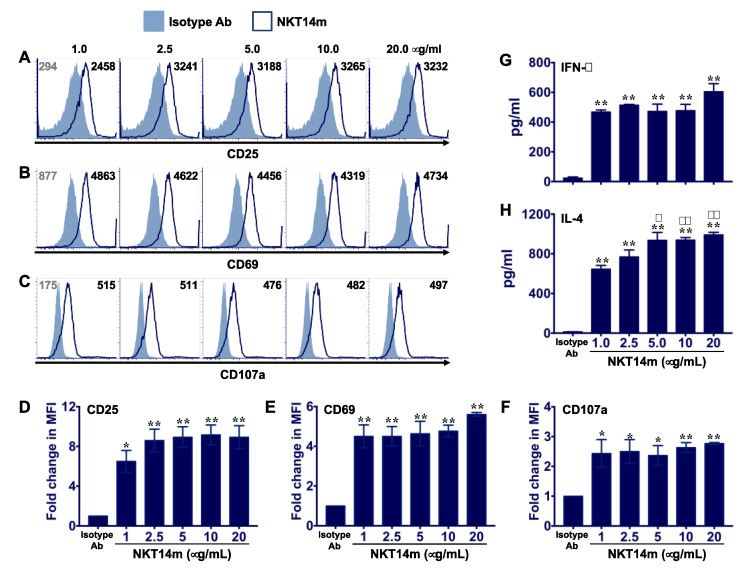
**NKT14m promotes iNKT cell activation and cytokine production in vitro.** (**A**–**H**) Freshly sorted liver iNKT cells from B6 mice were incubated with isotype control (20 µg/mL) or increasing concentrations of plate-bound NKT14m mAb as indicated. After 24 h, cells were analyzed for activation markers CD25 (**A**) and CD69 (**B**) and degranulation (CD107a, (**C**)) using flow cytometry. Data in (**A**–**C**) are representative from one of three independent experiments. Numbers in the histograms indicate mean fluorescence intensity (MFI). Compiled data (mean ± SEM) from three independent experiments showing fold increase in MFI for CD25 (**D**), CD69 (**E**) and CD107a (**F**) expression on iNKT cells plated on immobilized NKT14m as compared to cells plated on isotype control antibody. (**G**,**H**) Culture supernatants were harvested and analyzed for cytokines using ELISA. Data are presented as mean ± SEM from three independent experiments. Statistical significance in (**D**–**H**) was determined using one-way ANOVA (Tukey’s multiple comparison test). For each of the analyses, the mean of each group was compared to the mean of every other group. * *p* < 0.05, ** *p* < 0.01: isotype vs. all the other groups. # *p* < 0.05, ## *p* < 0.01: 1.0 µg/mL vs. all the other groups plated on immobilized NKT14m.

To test whether NKT14m can directly activate murine iNKT cells, we cultured sort-purified B6 liver iNKT cells on varying concentrations (1–20 µg/mL) of plate-bound NKT14m monoclonal antibody (mAb). Consistent with our recent studies using NKTT320 [28], we observed that immobilized NKT14m induced potent iNKT cell activation as demonstrated by increased surface expression of activation markers CD25 and CD69 (Figure 1A,B) as compared to cells plated on isotype control antibody. Invariant NKT cells predominantly mediate their direct cytolytic activity against tumor cells via the release of lytic molecules such as perforin and granzyme [24]. This release of lytic molecules onto the surface of target cells, also known as degranulation and a hallmark of cytotoxic lymphocyte activation, results in the externalization of CD107a (lysosome-associated membrane protein 1) on the cell surface. To determine whether NKT14m promotes iNKT cell degranulation, we plated purified murine iNKT cells on immobilized mAb in the presence of CD107a antibody. We observed that NKT14m induced a robust increase in the expression of CD107a (Figure 1C), suggesting that it can promote direct iNKT cell cytotoxicity. For each of these analyses (Figure 1D–F), we observed a vigorous response at the lowest concentration (1 µg/mL) of NKT14m evaluated, which did not increase significantly upon exposure to higher concentrations of the mAb. Finally, analysis of culture supernatants revealed that NKT14m induced abundant secretion of both IFN-γ (G) and IL-4 (H) that increased only marginally with higher concentrations of NKT14m, specifically for IL-4. Given that the viability of these cells was maintained at more than 90% (94 ± 1.5%) even at highest concentration of the mAb tested (Supplementary Figure S1), one possibility for the lack of a dose-dependent increase is that the iNKT cell response is saturated at the 1 µg/mL concentration. Nonetheless, these results provide direct evidence that NKT14m can efficiently engage the invariant TCR of iNKT cells and promote their activation and function, consistent with prior studies [29].

### 3.2. Invariant NKT Cells Readily Produce Cytokines in Response to NKT14m In Vivo

**Figure 2:**
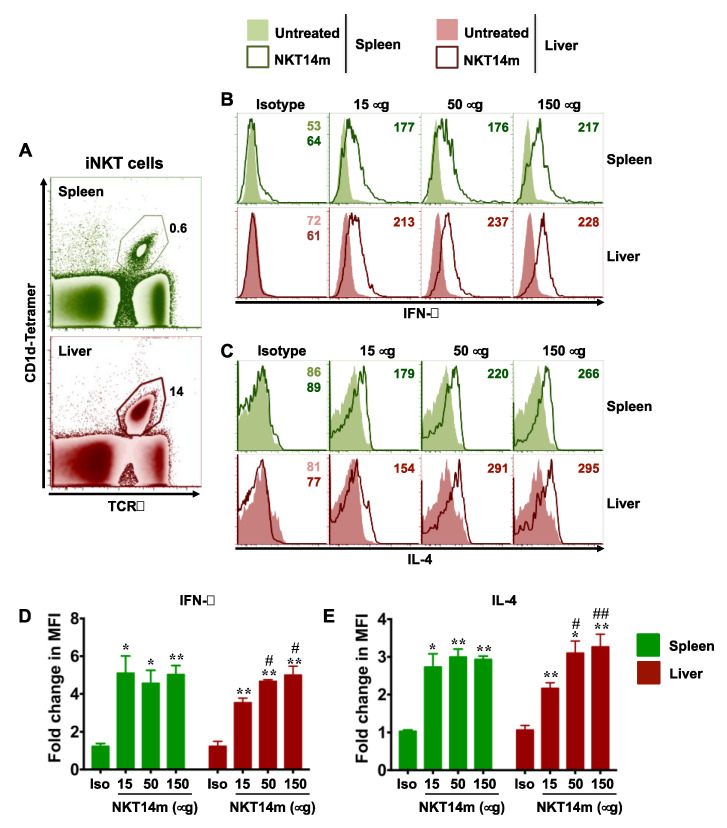
**NKT14m induces iNKT cell cytokine production in vivo.** (**A**–**E**) B6 mice were injected intravenously (i.v.) with different doses of NKT14m, 150 µg of isotype Ab or left untreated. After 2 h, the percentages of spleen and liver iNKT cells (as gated in (**A**)) producing IFN-γ (**B**) and IL-4 (**C**) directly ex vivo were analyzed using intracellular cytokine staining and flow cytometry. Data in (**B**) and (**C**) are from one of three independent experiments. Numbers in the histograms indicate MFI. (**D**,**E**) Pooled data (mean ± SEM) from three independent experiments showing fold change in MFI for IFN-γ (**D**) and IL-4 (**E**) expression in iNKT cells, as indicated in the graphs. Fold change in MFI was calculated as the ratio of MFI for each group to the MFI in uninjected mice. For each organ, statistical significance was determined using one-way ANOVA (Tukey’s multiple comparison test), where the mean of each group was compared to the mean of every other group. * *p* < 0.05, ** *p* < 0.01: isotype control (Iso) vs. all the other groups. # *p* <0.05, ## *p* < 0.01: 15 µg vs. 50 µg and 150 µg.

To characterize the effect of NKT14m on iNKT cell activation and functional response in vivo, we injected wild-type B6 mice with varying concentrations of NKT14m (15–150 µg) or isotype control antibody (150 µg) and 2 h later examined splenic and intrahepatic iNKT cell (Figure 2A) cytokine production (Figure 2B–E). Consistent with its inability to activate iNKT cells in vitro, the isotype control antibody failed to induce an in vivo iNKT cell response, even at the highest dose (150 µg). In contrast, in vivo administration of NKT14m readily mediated robust production of IFN-γ and IL-4 by splenic and hepatic iNKT cells at all the doses tested (Figure 2B–E). Although we did not observe any NKT14m dose-dependent increase in splenic iNKT cell IFN-γ or IL-4 levels (Figure 2D,E), there was a significant increase in the intracellular measure of these cytokines in liver iNKT cells, relative to both the isotype control antibody and the 15µg dose (Figure 2D,E).

### 3.3. NKT14m Induces Murine iNKT Cell Activation and Immunomodulatory Functions In Vivo

**Figure 3:**
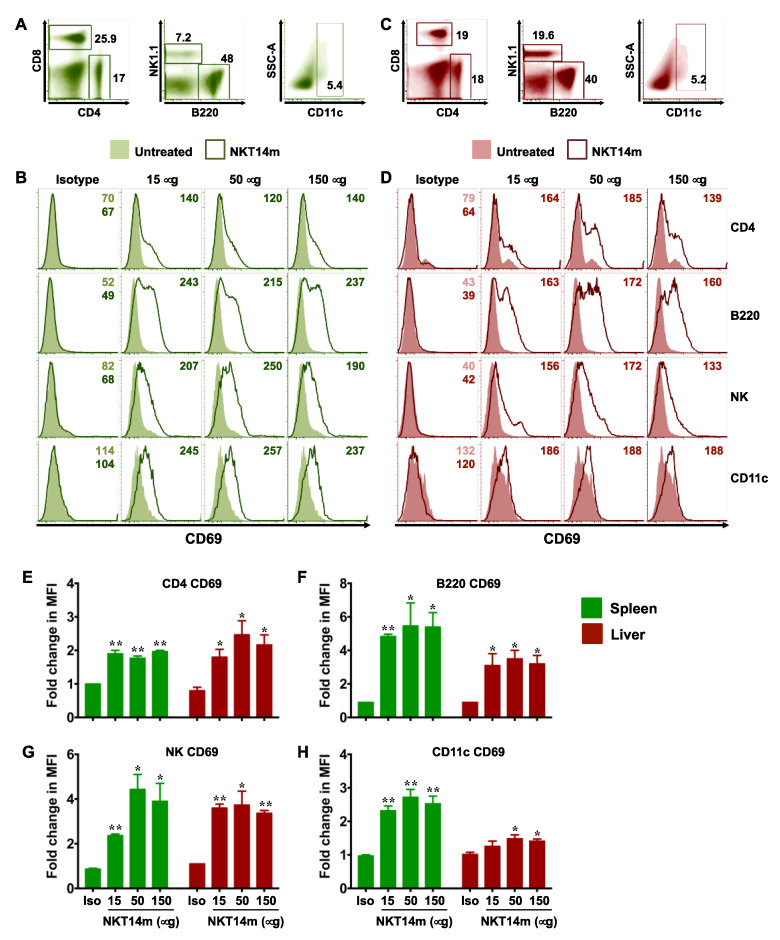
**NKT14m promotes iNKT cell-mediated transactivation of other immune cells in vivo.** (**A**–**H**) B6 mice were injected i.v. with different doses of NKT14m, 150 µg of isotype Ab or left untreated. After 6 h, splenocytes (**A**,**B**) and liver lymphocytes (**C**,**D**) were analyzed for CD69 expression (**B**,**D**) on CD4^+^, NK1.1^+^, B220^+^ and CD11c^+^ cells (as gated in (**A**,**C**)). Representative histograms (with MFI) from one of three independent experiments are shown. (**E**–**H**) Pooled data (mean ± SEM) from three independent experiments showing fold change in MFI for CD69 on various immune cells, as indicated in the graphs. Fold change in MFI was determined by dividing the MFI for each group by the MFI in uninjected mice. For each organ, statistical significance was determined using one-way ANOVA (Tukey’s multiple comparison test), where the mean of each group was compared to the mean of every other group. * *p* < 0.05, ** *p* < 0.01: isotype control (Iso) vs. all the other groups.

**Figure 4:**
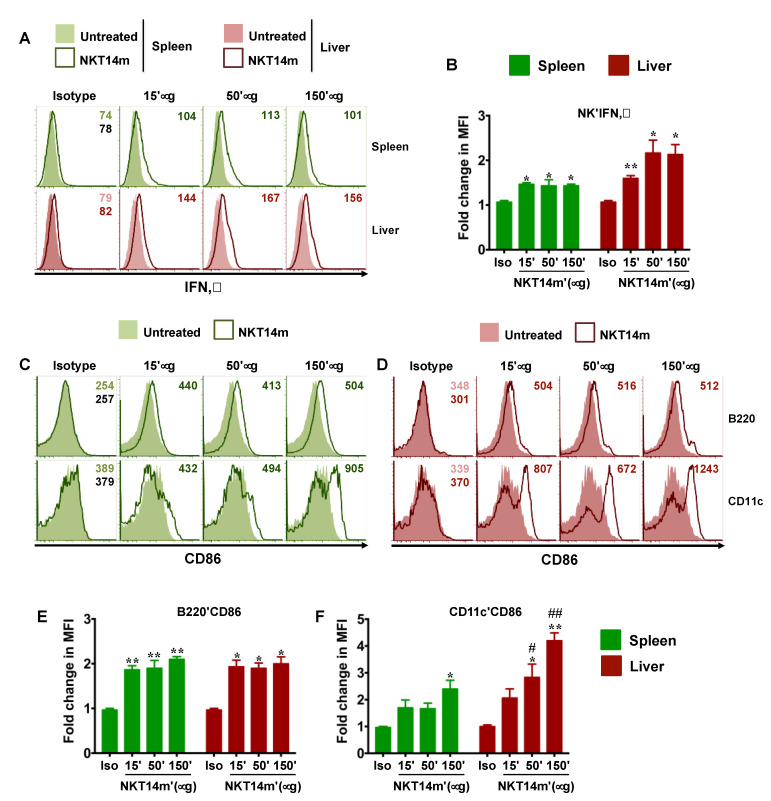
**NKT14m induces IFN-γ production by NK cells and upregulates CD86 on antigen presenting cells in vivo.** (**A**–**F**) B6 mice were injected i.v. with different doses of NKT14m, 150 µg of isotype Ab or left untreated. After 6 h, spleen and liver NK cells (as gated in Figure 3A,C) producing IFN-γ (**A**,**B**) directly ex vivo were analyzed using intracellular cytokine staining and flow cytometry. Representative histograms (with MFI) from one of three independent experiments are shown in (**A**). Mean fold change in MFI ± SEM from three independent experiments is shown in B (**C**–**F**). Spleen (**C**) and liver (**D**) B220^+^ and CD11c^+^ cells (as gated in Figure 3A,C) were analyzed for surface expression of CD86. Data in (**C**) and (**D**) are from one of three independent experiments and numbers in the histograms indicate MFI. Mean fold change in MFI ± SEM for CD86 (**E**,**F**) was pooled from three independent experiments. Fold change in MFI was calculated as the ratio of MFI for each group to the MFI in uninjected mice. For each organ, statistical significance was determined using one-way ANOVA (Tukey’s multiple comparison test), where the mean of each group was compared to the mean of every other group. * *p* < 0.05, ** *p* < 0.01: isotype control (Iso) vs. all the other groups. # *p* < 0.05, ## *p* < 0.01: 15 µg vs. 50 µg and 150 µg.

**Figure 5:**
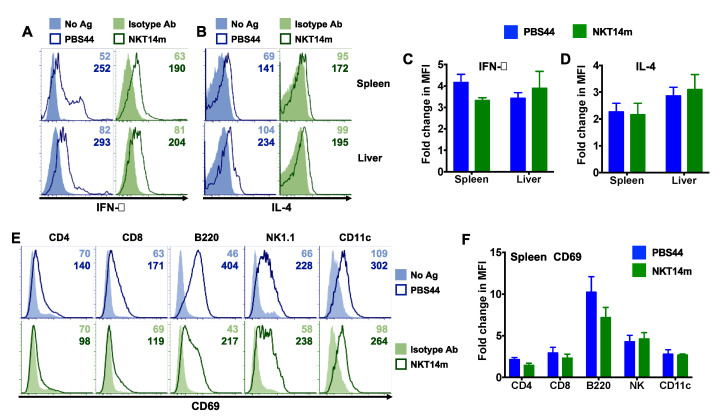
NKT14m activates murine iNKT cell functions in vivo similar to PBS44. (**A**–**F**) B6 mice were injected intraperitoneally (i.p.) with PBS44 (4 µg), NKT14m (i.v.) or isotype control antibody (50 µg, i.v.) or left untreated (No Ag). After 4–6 h, splenic and hepatic iNKT cells (as gated in Figure 2A) producing IFN-γ (**A**) or IL-4 (**B**) directly ex vivo were analyzed using intracellular cytokine staining and flow cytometry. Data in (**A**) and (**B**) are from one of three independent experiments. Numbers in the histograms indicate MFI. (**C**,**D**) Pooled data (mean ± SEM) from three independent experiments showing fold change in MFI for IFN-γ (**C**) and IL-4 (**D**) expression in iNKT cells, as indicated in the graphs. (**E**) Splenocytes were analyzed for CD69 expression on CD4^+^ CD8^+^, NK1.1^+^, B220^+^ and CD11c^+^ cells (as gated in Figure 3A) using flow cytometry. Representative histograms (with MFI) from one of three independent experiments are shown. Mean fold change in MFI ± SEM for CD69 (**F**) was pooled from three independent experiments. Fold change in MFI for IFN-γ (**C**), IL-4 (**D**) and CD69 (**F**) was calculated as the ratio of MFI in mice that received either PBS44 or NKT14m to the MFI in the respective control mice. Statistical significance in (**C**–**F**) was determined using an unpaired *t*-test with Welch’s correction. For each analysis in (**C**), (**D**) and (**F**), the mean fold change in MFI was compared between mice injected with PBS44 to those injected with NKT14m.

Once activated, iNKT cells serve to mature DCs and promote the functions of NK, T and B cells [31]. We next examined whether NKT14m enables activation of other immune cell lineages in vivo. To that end, mice were injected with varying concentrations (50–150 µg) of a single dose of NKT14m or the isotype control (150 µg) antibody. After 6 h, animals were euthanized and examined for up-regulation of CD69 on splenic and hepatic lymphocytes and myeloid cells (Figure 3A–H), IFN-γ production by splenic and hepatic NK cells (Figure 4A,B) and CD86 expression on antigen presenting cells (APCs, Figure 4C–F). We observed that mice receiving varying concentrations of the NKT14m antibody exhibited a dramatic increase in CD69 expression on T, B, NK and DCs in the spleen (Figure 3B) and the liver (Figure 3D), while those receiving isotype control antibody exhibited no response. Consistently, the fold change in MFI for CD69 was significantly higher at all the doses of NKT14m (compared to isotype control), both in the spleen and the liver immune cells (Figure 3E–H). Similarly, NK cells and APCs had increased intracellular IFN-γ (Figure 4A,B) and surface CD86 (Figure 4C–F) expression, respectively, in NKT14m but not isotype control antibody-treated mice. Importantly, in each of these analyses, NKT14m-mediated responses were comparable to those observed in mice receiving a single dose of the iNKT cell agonist, PBS44, in vivo (Figure 5A–F).

**Figure 6:**
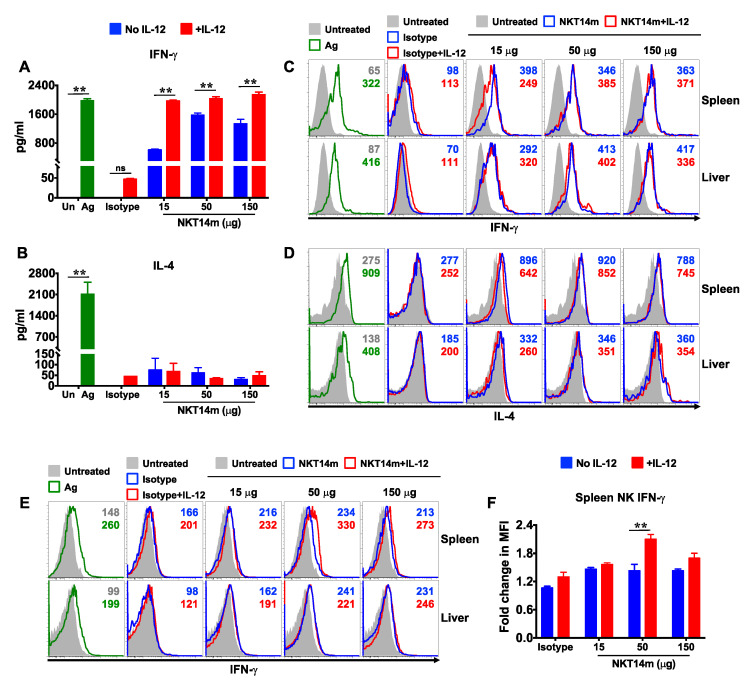
**IL-12 augments NKT14m-induced cytokine production in vivo.** (**A**–**F**) B6 mice were injected i.p. with antigen (Ag: PBS44, 4 µg) and were treated or not with IL-12 (i.p.) and different doses of NKT14m (i.v.), 150 µg of isotype Ab (i.v.) or left untreated (Un). After 4–6 h, serum was collected and IFN-γ (**A**) and IL-4 (**B**) levels were measured using ELISA. Data represent the mean ± SEM from three independent experiments. Significance was determined using an unpaired *t*-test with Welch’s correction. ** *p* < 0.01. ns: not significant. (**C**–**F**) Splenic and hepatic iNKT (**C**,**D**) and NK (**E**) cells (as gated in Figure 2A and Figure 3A, respectively) producing IFN-γ (**C**,**E**) or IL-4 (**D**) directly ex vivo were analyzed using intracellular cytokine staining and flow cytometry. Data in (C‒E) are from one of three independent experiments. Numbers in the histograms indicate MFI. (**F**) Compiled data (mean ± SEM) from three independent experiments showing fold change in MFI for IFN-γ expression in splenic NK cells, as indicated in the graph. Fold change in MFI was calculated as the ratio of MFI for each group to the MFI in uninjected mice. For each group, statistical significance was determined using two-way ANOVA (Sidak’s multiple comparison test); mean fold change in MFI without IL-12 was compared to the mean fold change in MFI with IL-12. ** *p* < 0.01.

Finally, we examined the serum cytokine levels in mice treated with PBS44 or NKT14m. Consistent with data shown in Figure 5, PBS44-induced iNKT cell activation correlated with significantly increased serum IFN-γ and IL-4 levels (Figure 6A,B). Similarly, NKT14m but not the isotype antibody was associated with an increase in serum IFN-γ levels (Figure 6A). In contrast, while NKT14m and PBS44 induced almost similar intracellular IL-4 production by hepatic and splenic iNKT cells (Figure 5B,D), the serum IL-4 levels in the presence of NKT14 were barely detectable (Figure 6B). These latter findings suggest that NKT14m preferentially promotes robust IFN-γ (Th-1) but not IL-4 (Th-2) responses in vivo. Collectively, these cellular and cytokine data (Figure 2, Figure 3, Figure 4, Figure 5 and Figure 6) reveal that NKT14m fosters rapid and robust iNKT cell-mediated immune system activation.

### 3.4. IL-12 Augments NKT14m-Induced Cytokine Production In Vivo

IL-12 promotes a strong anti-tumor response in several preclinical tumor models [32], and studies have indicated that iNKT cells are required for IL-12-induced anti-tumor responses [21,33]. We therefore examined the effect of IL-12 on NKT14m-induced iNKT cell activation in vivo. We observed that iNKT cell intracellular (Figure 6C,D and Supplementary Figure S2A–D) and serum levels (Figure 6A,B) of IFN-γ and IL-4 in mice injected with isotype control antibody and IL-12 were similar to those in mice treated with isotype control antibody alone or left untreated, suggesting that IL-12 alone did not induce iNKT cell activation. In contrast, serum IFN-γ levels were significantly higher in mice that received combination treatment (NKT14m+IL-12), as opposed to those that were injected with the antibody alone (Figure 6A). Although this IL-12-dependent increase in IFN-γ response was observed at all the doses of the mAb tested, it was most striking at the lowest dose (15 µg) of NKT14m. Consistent with its known role to induce a Th-1 response, IL-12 alone or in combination with NKT14m failed to induce IL-4 production (Figure 6B). Previously, it has been shown that IL-12 can increase the production of IFN-γ by activated iNKT cells [34]. However, we observed that iNKT cell intracellular levels of IFN-γ were comparable in mice that received either a single dose of NKT14m or a combination of NKT14m and IL-12 (Figure 6C and Supplementary Figure S2). Other cellular targets of IL-12 that can be induced to secrete IFN-γ include NK cells. Accordingly, we observed that NKT14m-mediated IFN-γ production by splenic (but not hepatic) NK cells was increased by IL-12, which was most significant at 50 µg of NKT14m (Figure 6E,F and Supplementary Figure S2E).

### 3.5. Treatment with NKT14m and IL-12 Controls Tumor Growth In Vivo.

**Figure 7:**
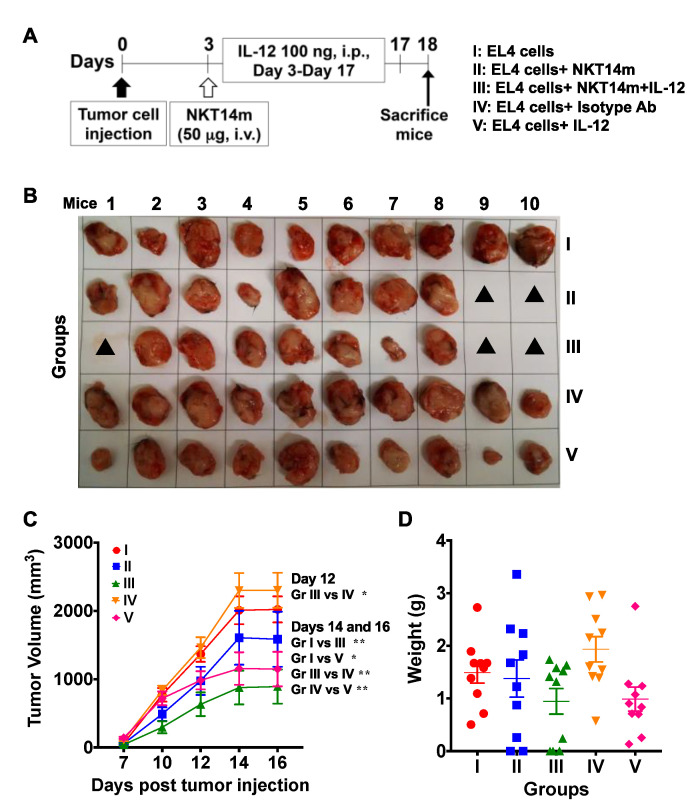
Combination treatment consisting of NKT14m and intraperitoneal injection of IL-12 has limited anti-tumor efficacy in vivo. Mice were injected with 0.3 × 10^6^ EL4 T lymphoma cells sub-cutaneously (s.c) in the right flank. After 3 days, mice in group II received a single dose (50 µg) of NKT14m antibody intravenously (i.v.) whereas mice in group III were injected with a single dose of NKT14m (50 µg, i.v.) and an intraperitoneal (i.p.) injection of 100 ng of IL-12. Mice in groups IV and V were injected with isotype control antibody (i.v.) and 100 ng of IL-12 alone, respectively. Mice in groups III and V received IL-12 daily from day 3 until the end of the experiment. Mice in the control group (group I) received neither NKT14m nor IL-12. Mice were euthanized on day 18 post tumor cell injection when several mice were moribund. (**A**) Schematic for experimental design. (**B**) Images of excised tumors. Closed black triangles indicate no visible or palpable tumors. (**C**) Tumor progression and (**D**) weight of excised tumors. Data in (**C**) and (**D**) are presented as mean ± SD of 10 mice per group from one independent experiment. Significance in (B) was determined using two-way ANOVA with Tukey’s multiple comparison test and in (D) using one-way ANOVA. * *p* < 0.05, ** *p* < 0.01.

To determine whether NKT14m is effective at controlling the growth of tumor cells in vivo, B6 mice were injected sub-cutaneously (s.c.) with EL4 T lymphoma cells. Three days post tumor cell injection; mice were left untreated or received a single injection of NKT14m or isotype control antibody. To test the anti-tumor effects of IL-12 in our model, we injected a cohort of mice with either IL-12 alone or in combination with NKT14m (Figure 7A). These animals received a daily injection of IL-12 starting day 3 until they were euthanized. Consistent with our in vitro and in vivo data, mice treated with the isotype control antibody (group IV, Figure 7B,C) were unable to inhibit tumor growth, similar to animals that received no mAb (group I, Figure 7B,C). Similarly, tumor-bearing mice treated with a single dose of NKT14m (group II, Figure 7B,C) failed to control tumor growth. Although mice that received IL-12 alone exhibited some reduction in tumor volume (group V, Figure 7B,C), the maximal control of tumor growth was observed in animals that received both NKT14m and IL-12 (group III, Figure 7C). However, there was no significant difference in the mean weight of the excised tumors between any groups (Figure 7D). 

**Figure 8:**
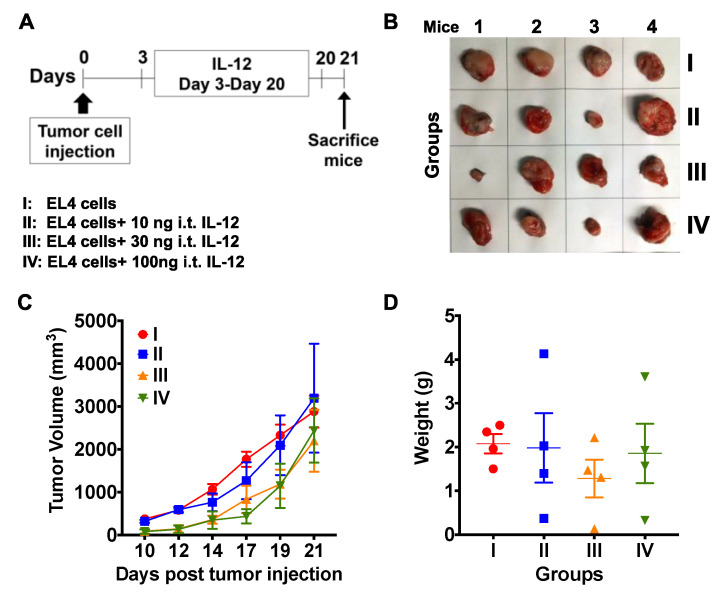
**Intra-tumor injection of IL-12 alone does not promote anti-tumor in vivo.** (**A**–**D**) Mice were injected s.c. with 0.3 × 10^6^ EL4 T lymphoma cells in the right flank. Mice in groups II‒IV received varying amounts (10–100 ng) of IL-12 i.t. daily from day 3 until the end of the experiment at three weeks. Control mice (group I) did not receive any IL-12. (**A**) Schematic, (**B**) images of excised tumors, (**C**) tumor progression and (**D**) weight of the excised tumors. Data in (**C**) and (**D**) are presented as mean ± SD of 4 mice per group from one of two independent experiments. Significance in (**C**) and (**D**) was determined using two-way and one-way ANOVA, respectively.

**Figure 9:**
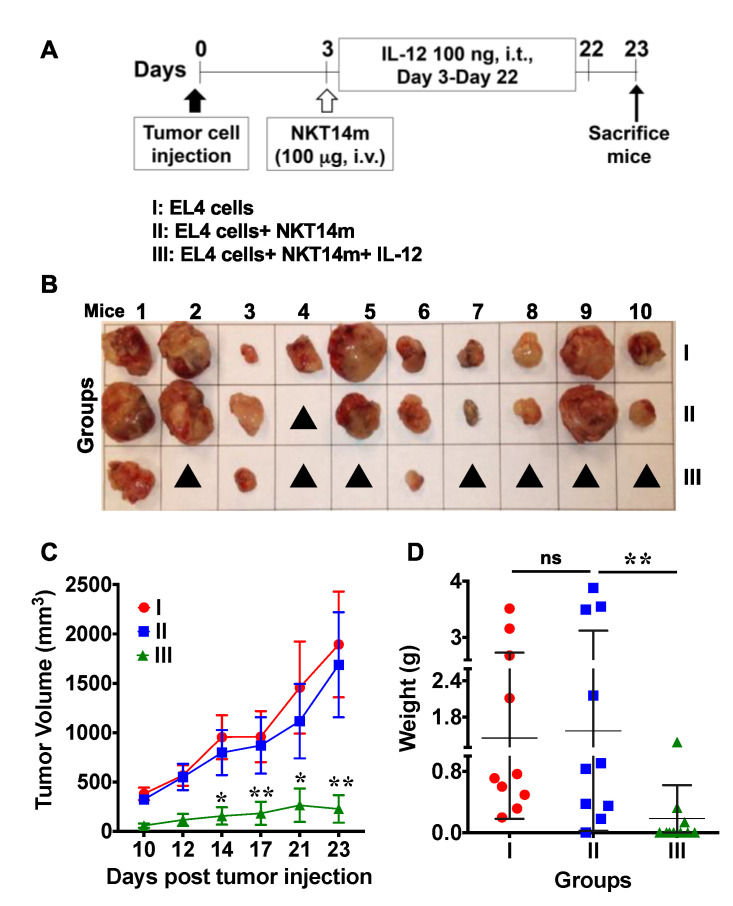
**Intra-tumor injection of IL-12 augments the anti-tumor activity of NKT14m in vivo**. Mice were injected s.c. with 0.3 × 10^6^ EL4 T lymphoma cells in the right flank. After 3 days, mice in group II received a single dose (100 µg) i.v. of NKT14m antibody, whereas mice in group III were injected with a single dose of NKT14m (100 µg, i.v) and intra-tumor (i.t.) injections of 100 ng of IL-12 daily (from day 3 onwards until the end of the experiment). Mice in the control group (group I) received neither NKT14m nor IL-12. (**A**) Schematic and (**B**) images of excised tumors from one representative experiment. Closed black triangles indicate no visible or palpable tumors. (**C**) Tumor progression and (**D**) weight of the excised tumors. Data in (**C**) and (**D**) are presented as mean ± SD of 10 mice per group from one independent experiment. Significance in (C) was determined using two-way ANOVA with Tukey’s multiple comparison test and in (D) using one-way ANOVA. * *p* < 0.05, ** *p* < 0.01, ns: not significant.

Prior studies have shown that IL-12 elicits a more potent anti-tumor response when existent in the tumor microenvironment rather than present systemically [35,36]. Thus, we examined the effect of intra-tumor (i.t.) injection of IL-12 on tumor progression. EL4 T lymphoma cells were injected s.c. into the right flank of mice, and 3 days later all the animals except for those in the control group received daily injections of varying amounts of IL-12 until the end of the study (Figure 8A–D). Initially (day 3), IL-12 was administered at the same site where the tumor cells were injected, and as the tumors developed and were palpable, IL-12 was injected directly into the tumors. Tumors in none of the groups responded to i.t. IL-12 therapy alone, as indicated by continued tumor growth (Figure 8C) and increased the final size and weight of the tumors (Figure 8B,D). In contrast, we observed significant control of tumor growth following treatment with NKT14m along with daily i.t. injections of IL-12 (group III, Figure 9A–D). Mice in this group either had very small tumors that were less than 300 mm^3^ or had no visible or palpable tumors at all. Altogether, these latter data identify NKT14m as a novel stimulatory antibody that can be effectively used in conjunction with IL-12 to augment the anti-tumor functions of iNKT cells. Importantly, these results also indicate that the route of IL-12 administration is critical in determining the outcome of the anti-tumor response.

## 4. Discussion

Cancer immune surveillance involves a complex interplay between transformed, tumor-supporting stromal and immune cells. While the contributions of CD8^+^T and NK cells to anti-tumor immunity are well-appreciated [37], mounting evidence also supports the development of innovative iNKT cell-based therapies for the treatment of cancer [38,39,40]. Invariant NKT cells are an attractive candidate for cancer immunotherapy as they can exert robust lysis of tumor cells as well as stimulate the anti-tumor functions of other cells of the immune system [38]. Furthermore, the limited polymorphism of the human CD1d gene and lack of CD1d-incompatibility between donors and recipients renders transfer of mature iNKT cells a safer approach than the infusion of conventional T cells. However, to capitalize on the potential benefits of iNKT cell-based cancer therapies, it is critical that we develop new strategies to enhance their anti-tumor function. To that end, we recently characterized the iNKT cell agonistic properties of NKTT320, a novel recombinant humanized monoclonal antibody (mAb) that binds selectively and with high affinity to the human iTCR [28]. Strikingly, NKTT320 induced robust human iNKT cell activation, proliferation and cytokine production. Human iNKT cells stimulated by NKTT320 upregulated cytotoxic markers, such as granzyme B, and exhibited robust degranulation. Importantly, NKTT320 also induced dramatic iNKT cell-mediated transactivation of other immune cells, suggesting that iNKT cell TCR-specific mAbs can facilitate both direct and indirect iNKT cell cytotoxicity.

To test this directly in an in vivo tumor model, in the current study we used NKT14m, a recently developed and tested monoclonal antibody specific for the TCR of murine iNKT cells [29,30]. We observed that administration of a single dose of NKT14m had limited efficacy in controlling tumor growth in vivo (Figure 7 and Figure 9). This was rather surprising because a single dose of NKT14m induced robust iNKT cell activation in vitro and in vivo (Figure 1 and Figure 2) as well as transactivation of several immune cells in vivo (Figure 3 and Figure 4). Furthermore, mice injected with NKT14m exhibited high serum IFN-γ levels but had barely any IL-4, suggesting that NKT14m supported an inflammatory Th1 cytokine response (Figure 6A,B). Thus, one possibility for the poor anti-tumor response by NKT14m in tumor-bearing mice could be the immunosuppressive effect of the tumor microenvironment (TME), as is often the case in solid tumors [41]. One approach to revert this immune suppression within the TME is by intra-tumor (i.t.) injection of cytokines that can initiate and sustain an inflammatory response. Toward this end, IL-12 is a potent immunostimulatory cytokine that activates the innate and adaptive immune system and exhibits pleiotropic effects, including maturation of antigen presenting cells and activation of NK and CD8^+^T cells, enhancing IFN-γ secretion, promoting generation of CD4^+^ Th1 cells and recalibrating myeloid derived suppressor cells [32,42,43]. In agreement with these roles, IL-12 has shown significant therapeutic effect in several preclinical tumor models [32,42,43]. However, such activity depends on the tumor type, dose and route of IL-12 injection [32]. Additionally, studies have implicated that iNKT cells are essential in IL-12-induced anti-tumor responses as iNKT-deficient mice cannot mediate IL-12-induced rejection of FBL-3 erythroleukemia or B16 melanoma tumors [21,33]. Conversely, activation of iNKT cells by αGC requires both endogenous IL-12 produced by DCs as well as direct contact of DCs and iNKTs via CD40/CD40 L interaction [44]. 

In our study, we observed that low dose IL-12 by itself failed to promote tumor regression, consistent with its inability to stimulate iNKT cell activation or IFN-γ production (Figure 6). In support of this, other groups have shown that monotherapy of IL-12 has limited therapeutic effects [32]. However, given its compelling stimulatory effects on various immune cells, it is a promising candidate for use in combination with other therapeutic agents [42,43]. Indeed, we demonstrated that when administered in conjunction with NKT14m, IL-12 significantly increased IFN-γ production in vivo (Figure 6A). Accordingly, combination treatment consisting of a single dose of NKT14m and low dose IL-12 significantly reduced tumor size and morbidity in mice engrafted with T-lymphoma cells (Figure 9). Although the exact cellular mechanism underlying this anti-tumor response remains to be elucidated, it is tempting to speculate that a synergism of iNKT and NK cells exists, as both respond to IL-12 by rapidly releasing IFN-γ [45]. This could also explain the negligible difference in intracellular levels (Figure 6C–F) but higher serum IFN-γ (Figure 6A) in mice injected with NKT14m+IL12 as compared to those treated with NKT14m alone. We also observed an upregulation of the activation marker (CD69) and co-stimulatory molecule (CD86) on CD11c^+^ DCs in mice injected with NKT14m. Thus, it also likely that in the presence of NKT14m and IL-12, coordinated interactions between iNKT cells and DCs (which rapidly produce IFN-γ in the presence of IL-12) facilitate tumor control by recruiting and activating downstream effectors including NK and cytotoxic CD8^+^T cells.

Importantly, i.t. injection of IL-12 induced a more efficacious anti-tumor response in tumor-challenged mice as compared to those that received systemic injections of IL-12 (Figure 7 and Figure 9). These data are in line with prior studies demonstrating that systemic treatment with IL-12 has no effect against subcutaneous growth of C26 colon carcinoma cells [46]. Furthermore, several studies have demonstrated that systemic administration of IL-12 is associated with severe toxicities that have greatly limited its clinical application [32]. A clear benefit of i.t. administration of IL-12 is that much higher local cytokine levels can be achieved that can promptly activate various immune cells to augment the anti-tumor response. Additionally, i.t. injection can potentially reduce IL-12 levels in the circulation, which could lessen the toxicity associated with systemic delivery of IL-12. Consistent with this notion, several recent preclinical and clinical studies have used tumor-infiltrating lymphocytes and CAR-T cells with constitutive or inducible IL-12 to localize the effects of IL-12 into the TME [35,36]. The anti-tumor effects mediated by local IL-12 secretion can also be enhanced by systemic administration of IL-18 [47]. Akin to IL-12, IL-18 has been shown to promote anti-tumor responses in several mouse models [48,49,50,51,52,53]. Specifically, systemic administration of IL-18 [48,49,50,51] or IL-18 gene therapy [52,53] abrogates tumor growth and prolongs the survival of tumor-bearing mice. Interestingly, IL-18 not only augments both NK and iNKT cell activity via IFN-γ production [54], but it also acts synergistically with IL-12 to enhance the cytotoxicity of these cells against tumor targets [55,56,57]. Indeed, adoptive transfer of iNKT cells activated in vitro with a combination of IL-12 and IL-18 robustly produces IL-2 and IFN-γ, which in turn activates NK cells to mediate anti-tumor response in vivo [34]. Although we have not yet formally tested the effects of IL-18 alone or IL-18+IL-12 in conjunction with NKT14m, future studies are warranted to explore these combination treatments to broaden the modalities for iNKT cell-based cellular immunotherapy for cancer.

In the last decade, studies from several laboratories including ours have designed and tested alternative approaches to enhance and sustain the anti-tumor activity of iNKT cells. These include chimeric antigen receptor (CAR)-therapy [58] and bi-specific fusion proteins [59,60,61]. While these approaches are promising, their wider therapeutic application and clinical success relies on the identification of tumor-associated antigens (TAA) that are exclusively expressed on tumors cells, have high immunogenicity and do not undergo immune editing. To override these limitations, agonistic anti-iTCR mAbs provide a viable alternative to target a wider array of cancers. Indeed, prior studies have established that iNKT cell functions can be manipulated by anti-iTCR mAbs for therapeutic purposes. For instance, NKTT120 [62,63], a humanized iTCR mAb that selectively depletes iNKT cells, has been developed for sickle cell disease [62]. Similarly, the activating (NKT14m) and depleting (NKT14) versions of the murine iTCR mAb can delay or accelerate the onset of Type 1 diabetes in NOD mice [29]. Furthermore, it has been recently shown that the activating NKT14m mAb, when administered alone or in combination with the chemotherapeutic agent cyclophosphamide to tumor-bearing mice, significantly increases iNKT cell anti-tumor response and prolongs survival [30]. In the same study, repeated injection of NKT14m did not induce anergy, a phenomenon that has restricted the clinical use of the iNKT cell agonist αGC [30]. Collectively, these studies, along with our recent report on NKTT320 mAb [28], warrant further development of anti-iTCR mAbs that can be used alone or in conjunction with cytokines (such as IL-12 and/or IL-18) or chemotherapeutic agents to reduce the toxicity associated with these agents while enhancing iNKT cell anti-tumor immunity against various cancers.

## Supplementary Materials

Supplementary materials can be found at https://www.mdpi.com/1422-0067/21/14/5085/s1.
