# Supplementary material for: Combination of NKT14m and Low Dose IL-12 Promotes Invariant Natural Killer T Cell IFN-γ Production and Tumor Control
Source: Int J Mol Sci. 2020 Jul 18;21(14):5085. doi: 10.3390/ijms21145085 (PMC7404385; doi:10.3390/ijms21145085)
Supplement: Supplementary file 1 [file ijms-21-05085-s001.pdf]

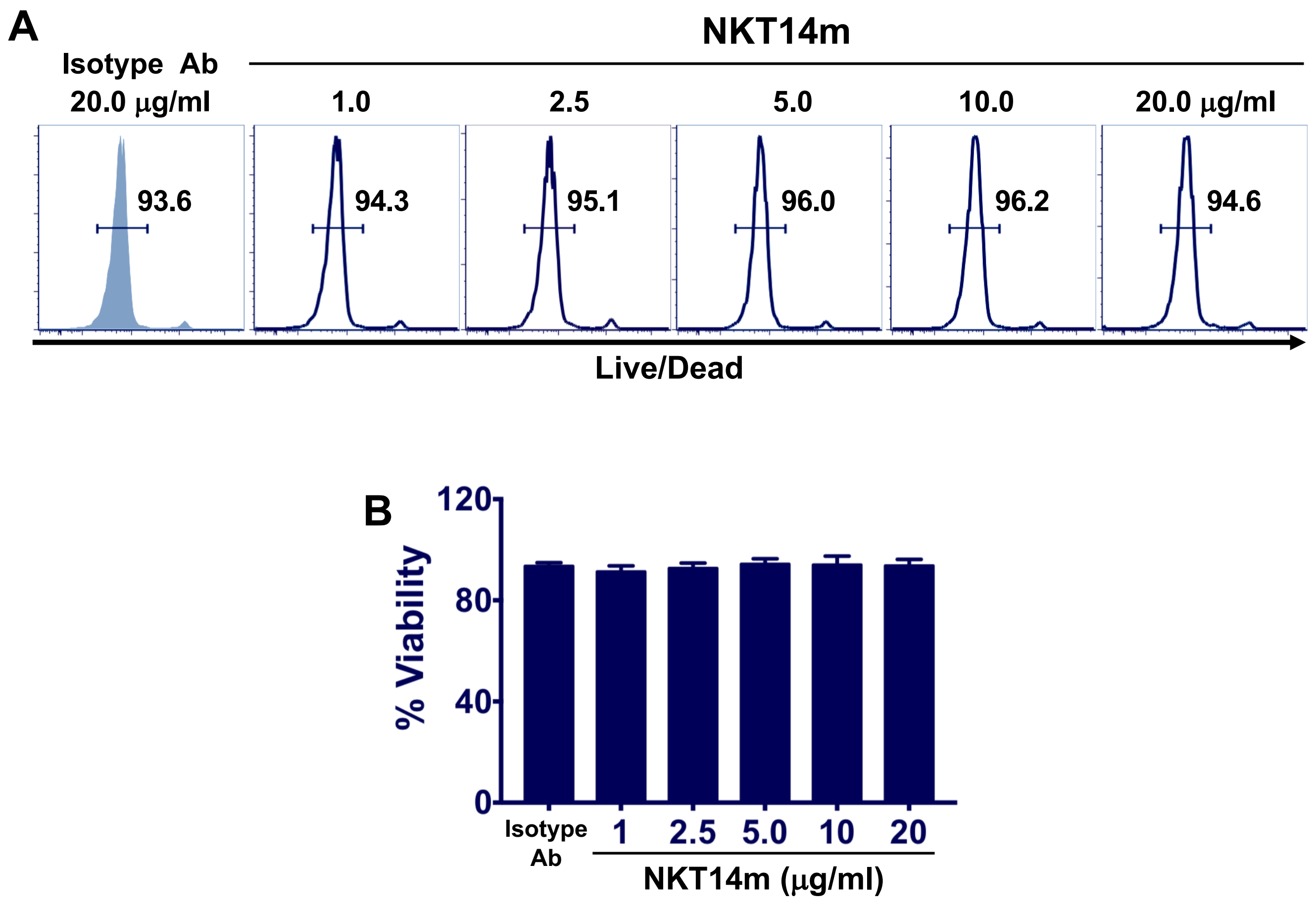

A
NKT14m
Isotype Ab
20.0 μg/ml
1.0
2.5
5.0
10.0
20.0 μg/ml
93.6
94.3
95.1
96.0
96.2
94.6
Live/Dead
B
% Viability
120
80
40
0
Isotype Ab
1
2.5
5.0
10
20
NKT14m (μg/ml)

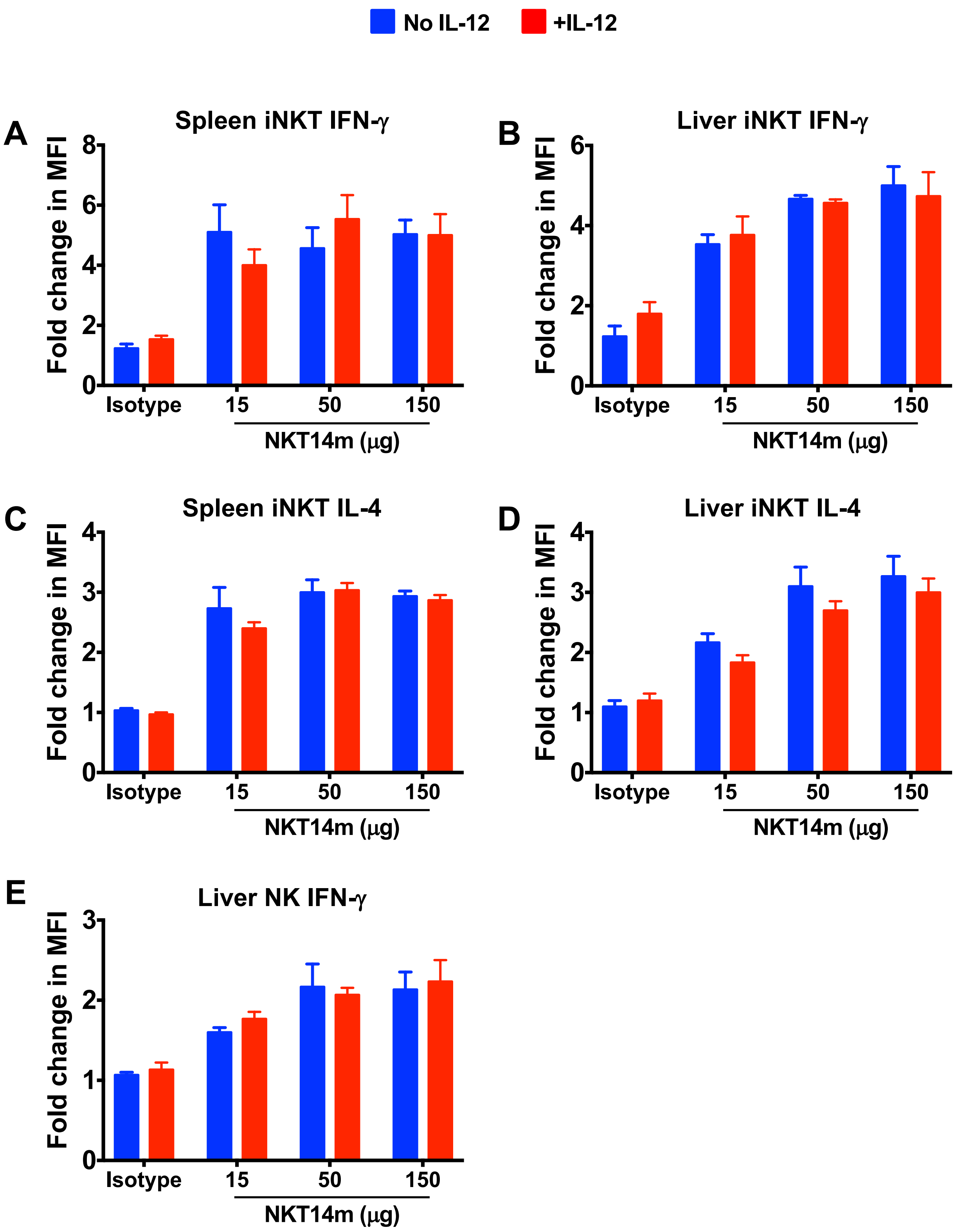
No IL-12
+IL-12
A
Spleen iNKT IFN-γ
B
Liver iNKT IFN-γ
C
Spleen iNKT IL-4
D
Liver iNKT IL-4
E
Liver NK IFN-γ
Fold change in MFI
Isotype
15
50
150
NKT14m (μg)

Supplementary Figure S1. NKT14m does not impair viability of iNKT cells. (A, B) Freshly sorted liver iNKT cells from B6 mice were incubated with isotype control (20 μg/mL) or increasing concentrations of plate-bound NKT14m mAb as indicated. After 24 h, cells were stained with LIVE/DEAD Fixable Aqua Dead Cell dye and analyzed for viability by flow cytometry. Representative histograms (A) from one of three independent experiments are shown. Numbers in the histograms indicate percent viable cells. (B) Compiled data are presented as mean percent viability ± SEM from three independent experiments.

Supplementary Figure S2. Intracellular IFN-γ levels in iNKT and liver NK cells are comparable in mice injected with NKT14m or NKT14m+IL-12. (A-E) B6 mice were injected i.p. with antigen (Ag: PBS44, 4 μg), were treated or not with IL-12 (i.p.) and different doses of NKT14m (i.v), 150 μg of isotype control Ab (i.v) or left untreated. After 4-6 h, the amount of IFN-γ or IL-4 produced directly *ex vivo* by splenic and liver iNKT cells and liver NK cells was analyzed by intracellular cytokine staining and flow cytometry. Compiled data (mean ± SEM) from three independent experiments showing fold change in MFI for IFN-γ (A, B, E) and IL-4 (C, D) expression in iNKT and NK cells, as indicated in the graph. Fold change in MFI was calculated as the ratio of MFI for each group to the MFI in uninjected mice. For each group, statistical significance was determined using two-way ANOVA (Sidak's multiple comparison test); mean fold change in MFI without IL-12 was compared to the mean fold change in MFI with IL-12.

Supplemental Table 1

Antigen	Fluorochrome	Catalog #	Clone	Company
CD11c	FITC	11-0114-82	N418	eBioscience
CD25	FITC	553072	7D4	BD Pharmingen
CD4	FITC	553047	RM4-5	BD Pharmingen
CD69	FITC	553236	H1.2F3	BD Pharmingen
CD8a	FITC	553031	53-6.7	BD Pharmingen
IFN-γ	FITC	554411	XMG1.2	BD Pharmingen
NK1.1	FITC	553164	PK136	BD Pharmingen
TCRβ	FITC	553171	H57-597	BD Pharmingen
CD107a (LAMP-1)	PE	558661	1D4B	BD Pharmingen
CD25	PE	553866	PC61	BD Pharmingen
CD4	PE	553730	GK1.5	BD Pharmingen
CD45R (B220)	PE	553090	RA3-6B2	BD Pharmingen
CD69	PE	553237	H1.2F3	BD Pharmingen
CD8a	PE	553033	53-6.7	BD Pharmingen
IFN-γ	PE	554412	XMG1.2	BD Pharmingen
IL-4	PE	554389	BVD4-1D11	BD Pharmingen
NK1.1	PE	108708	PK136	Biolegend
TCRβ	PE	553172	H57-597	BD Pharmingen
CD25	APC	557192	PC61	BD Pharmingen
CD4	APC	553051	RM4-5	BD Pharmingen
CD8a	APC	553035	53-6.7	BD Pharmingen
IL-4	APC	554436	11B11	BD Pharmingen
NK1.1	APC	550627	PK136	BD Pharmingen
TCRβ	APC	553174	H57-597	BD Pharmingen
CD8a	PerCP Cy5.5	551162	53-6.7	BD Pharmingen
IFN-γ	PerCP Cy5.5	45-7311-80	XMG1.2	eBioscience
NK1.1	PerCP Cy5.5	551114	PK136	BD Pharmingen
CD4	Pacific Blue	100428	GK1.5	Biolegend
TCRβ	Pacific Blue	109226	H57-597	Biolegend
CD86	PE-Cy7	560582	GL1	BD Pharmingen
